# Optimal Computational Modeling and Simulation of QCA Reversible Gates for Information Reliability in Nano-Quantum Circuits

**DOI:** 10.3390/nano14171460

**Published:** 2024-09-08

**Authors:** Jun-Cheol Jeon

**Affiliations:** Department of Convergence Science, Kongju National University, Gongju 32588, Republic of Korea; jcjeon@kongju.ac.kr

**Keywords:** nanotechnology, quantum-dot cellular automata, reversible gate, circuit reliability, information security

## Abstract

As the relationship between energy and information loss and reversible gates was revealed, much interest in reversible gate design arose, and as quantum-dot cellular automata (QCA) gained attention as a next-generation nano circuit design technology, various reversible gates based on QCA emerged. The proposed study optimizes the performance and design costs of existing QCA-based reversible gates including TR, RUG, PQR, and URG. According to most indicators, the proposed circuits showed significant improvement rates and outperformed existing studies. In particular, the proposed optimal TR, RUG, PQR, and URG showed performance improvements of 266%, 265%, 300%, and 144% in *Cost_AD_*, respectively, compared with the best existing circuit. This shows outstanding improvement and superiority in terms of area and delay, which are the most important factors in the performance of nano-scale circuits that are becoming extremely miniaturized. Additionally, the exceptionally high-output polarization of the proposed circuits is an important indicator of the circuit’s expansion and connection and increases the circuit’s reliability.

## 1. Introduction

In the 1960s, Landauer discovered that information deletion requires entropy [1], and, inspired by this, Bennett first proposed reversible computation in the 1970s [2]. Landauer demonstrated that the loss of one bit of information results in a certain energy loss, and Bennett proved the validity of this. This energy loss that occurs in an irreversible circuit can be recovered in a reversible circuit, and a direct relationship between information loss, energy dissipation, and reversible circuit can be confirmed [2].

In 1980, Toffoli proposed a reversible operation using the controlled-controlled NOT gate (CCNOT) [3], and in 1982, Fredkin and Toffoli proposed conservative logic and reversible computing to calculate the collision of billiard balls using this [4]. In 1985, Feyman proposed reversible computing using a controlled NOT gate (CNOT) [5], and Peres proposed a gate that could perform both CCNOT and CNOT operations simultaneously [6]. Later, based on this research, various academic theorems about quantum computing and various reversible gates appeared [7,8,9,10,11].

Meanwhile, quantum-dot cellular automata (QCA) were proposed by Tougaw and Lent in 1993 to overcome the problems of high power loss and information loss in existing CMOS circuits [12,13]. This technology has significant advantages, such as operating frequency (THz), high device density, and low power consumption [14]. The dissipated energy is measured based on the Hamiltonian matrix using the HartreeFock approximation related to the Coulomb repulsion between QCA cells [15]. The power dissipation using Hamiltonian matrix can be summarized in terms of energy per clock cycle, as shown in Equation (1) [16].

(1) $$P_{diss}=\frac{E_{diss}}{T_{c}}\langle \left.\frac{\hbar }{2T_{c}}{\vec{\Gamma }}_{+}\times \left[-\frac{{\vec{\Gamma }}_{+}}{\left|{\vec{\Gamma }}_{+}\right|}\tanh\left(\frac{\hbar \left|{\vec{\Gamma }}_{+}\right|}{k_{B}T}\right)+\frac{{\vec{\Gamma }}_{-}}{\left|{\vec{\Gamma }}_{-}\right|}\tanh\left(\frac{\hbar \left|{\vec{\Gamma }}_{-}\right|}{k_{B}T}\right)\right]\right.\rangle$$

where $T_{c}$ is the clock period and ${\vec{\Gamma }}_{+}$ and ${\vec{\Gamma }}_{-}$ are the Hamiltonian values before and after the transaction, *k*_B_ = 1.38 × 10^−23^ JK^−1^ is the Boltzmann constant, and *T* is the temperature in Kelvin.

With the rapid development of quantum computing, various studies are continuing to improve the implementation and efficiency of QCA-based reversible gates such as TR, RUGs, RQCA, PQR, and URGs. In this study, optimization was performed to improve the performance and design cost of QCA based reversible gates. The key contributions of this paper are as follows:

- an overview of previously proposed QCA-based reversible gates;
- performance optimization of QCA-based reversible gates such as TR, RUGs, PQR, and URGs;
- significant performance improvement in two representative design cost metrics related to area delay and energy delay;
- remarkable improvement in average output polarization required for reliability of circuit interconnection and expansion.

The remainder of the paper is organized as follows. Section 2 introduces existing QCA-based reversible gates and explains basic knowledge about QCA. In Section 3, we describe optimization of QCA-based reversible gate circuits and confirm their operation through simulation results. In Section 4, the proposed circuits are analyzed and compared with existing studies and various performance indicators and design costs are considered, and Section 5 concludes.

## 2. Related Works

### 2.1. Reversible Gates

**Figure 1:**
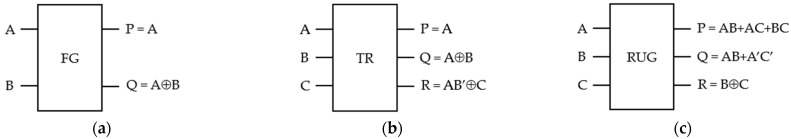

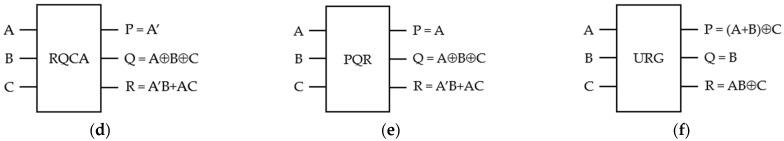
Block diagram of QCA based reversible gates with inputs (A, B, C) and outputs (P, Q, R): (**a**) FG; (**b**) TR; (**c**) RUG; (**d**) RQCA; (**e**) PQR; (**f**) URG.

The Feynman gate (FG), well known as the CNOT gate in Figure 1a, uses XOR as the basic operation in digital logic. This operation is used as a necessary operation in most reversible gates [5]. Figure 1b illustrates a TR gate designed by Thapliyal et al. who proposed a reversible binary subtractor, and the gate was designed to directly obtain the AB’ required for the half subtractor [7]. Figure 1c shows the RUG proposed by Sen et al., which eliminates garbage output and has been designed as an energy-efficient gate that can be used as a universal gate [8]. Sen et al. proposed an RQCA gate with excellent fault tolerance, as shown in Figure 1d, for the purpose of designing a reversible ALU (arithmetic and logic unit) [9]. The PQR gate in Figure 1e was proposed by Chabi et al. as a universal gate with low design cost, but did not show a design significantly different from RQCA [10]. Figure 1f presents the URG proposed by Islam et al. as a universal reversible gate that is efficient in energy and delay [11].

### 2.2. Background of QCA

**Figure 2:**
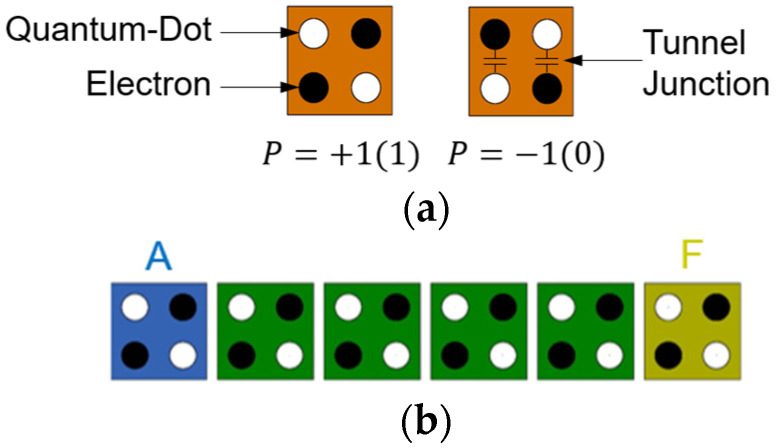
QCA cell: (**a**) two types of cell with different polarization; (**b**) QCA wiring.

Molecular quantum-dot cellular automata (QCA) represent a low-power computing paradigm that can provide ultra-high device density and THz speed switching at room temperature [17,18,19]. The QCA structure consists of a QCA cell, which has four quantum dots at the corners of a square, as shown in Figure 2a, and two electrons are always located on the diagonal due to Coulomb repulsion. Electrons can move through tunnels between quantum dots and have two polarizations: P = +1 and P = −1, corresponding to 1 and 0, respectively, in binary operation. Figure 2b shows the output F according to input A. When cells are connected in succession, they serve as a wire that transmits the same value [13,20].

**Figure 3:**
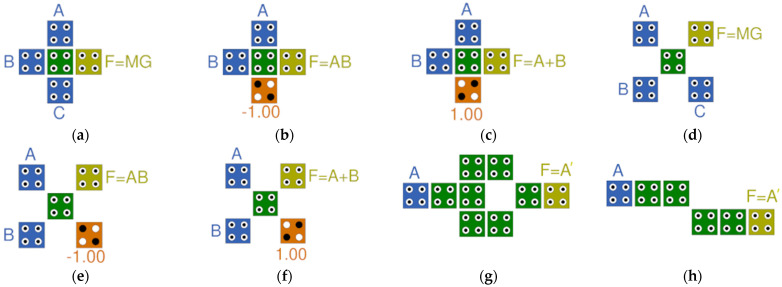
QCA basic logic gates: (**a**) majority gate with inputs (A, B, C) and output (F); (**b**) AND gate with fixed cell (P = −1); (**c**) OR gate with fixed cell (P = +1); (**d**) rotated majority gate; (**e**) rotated AND gate; (**f**) rotated OR gate; (**g**) robust NOT gate; (**h**) simple NOT gate.

The basic operations of QCA are operated by majority voting gates. Figure 3a,d show a majority vote and a rotated majority gate with three inputs (A, B, C) and one output F, respectively. These can function as AND gates by fixing one input to the value of −1, as shown in Figure 3b,e. Additionally, as shown in Figure 3c,f, one input can be fixed to the value of +1 to function as an OR gate. Figure 3g,h show a robust NOT gate and a simple NOT gate, respectively [12,21].

The operation and gate configuration within the circuit are made possible by the basic logic gates shown in Figure 3 but can be achieved by using gates created by cell interaction. For example, the two-input XOR gate is a complex two-level gate that requires two ANDs, two inverters, and one OR gate, but it can be effectively designed as a simple one-level gate constructed via cell interaction. Therefore, various cell-interaction-based gates have been developed recently to replace majority-gate-based gates [20,21].

**Figure 4:**
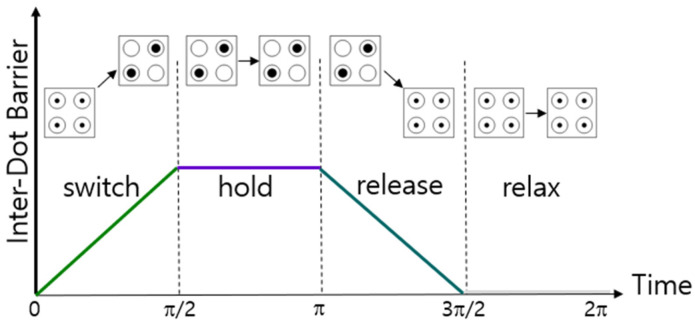
Change of four clock phases over time.

QCA operates by a clocking system as shown in Figure 4. In QCA, one clock cycle consists of four clock phases, which vary depending on the movement of electrons according to the height of the barrier between quantum dots. A switch refers to a state in which the barrier between quantum dots gradually increases, and a hold refers to a state in which the barrier between quantum dots becomes sufficiently high that electrons can no longer move and have a specific polarization. The state in which the barrier between quantum dots gradually lowers over time is called release, and the state in which the barrier is sufficiently lowered so that electrons can move freely is called relax. A QCA cell transmits and maintains values by repeating these four states [22,23].

## 3. Proposed Reversible Gates Based on QCA

**Figure 5:**
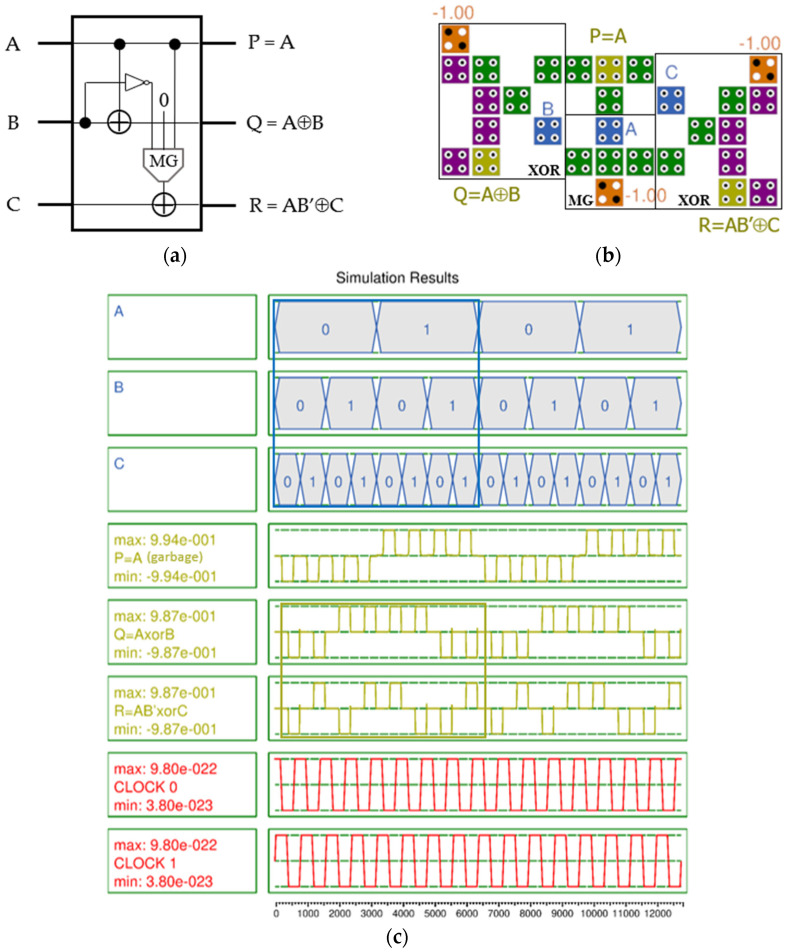
TR gate with inputs (A, B, C) and outputs (P, Q, R): (**a**) logic diagram; (**b**) QCA implementation; (**c**) simulation results.

Recently, various reversible gates have been designed using QCA. Therefore, in this section, optimization of the QCA-based reversible gate is described. In particular, we aimed to improve performance and lower design costs by efficiently implementing reversible gates such as TR, RUG, URG, and PQR. Figure 5a shows the logic diagram of the TR gate [7]. The first output P of the TR gate outputs the input value as is, and the second Q and third R output A⊕B and AB’⊕C, respectively. The proposed design uses the three-input XOR gate proposed in [24] and the modified two-input XOR gate. The proposed structure shown in Figure 5b was designed simultaneously using two inputs, B and B′, as a simple inverter for efficient design, and the result of AB′ was used as an input for output R without any delay. As a result, the proposed QCA-based TR gate requires 29 cells, an area of 25,272 nm^2^, and 0.5 clock cycles.

In the simulation results in Figure 5c, the blue box shows all changes in the input values, and the green box indicates the results of valid output values depending on the inputs, and the output polarization of both valid outputs Q and R shows a very high value of 0.987. Although there is a slight jump in the output signal, it is very stable overall and there is no signal distortion.

**Figure 6:**
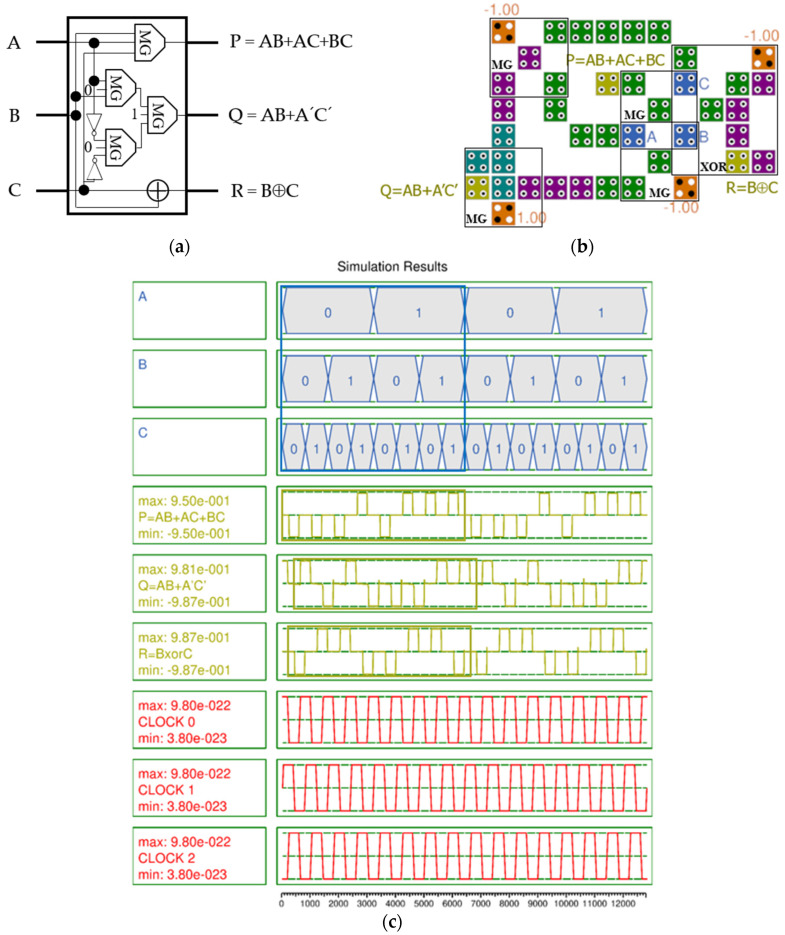
RUG with inputs (A, B, C) and outputs (P, Q, R): (**a**) logic diagram; (**b**) QCA implementation; (**c**) simulation results.

Figure 6a shows the logic diagram of the RUG [8]. P outputs the majority vote function of inputs A, B, and C, and Q and R output AB + A′C′ and B⊕C, respectively. Four majority gates are required to operate P and Q, and one two-input XOR gate is required for R. For design efficiency, inputs A and B included in the two MGs are shared, and inputs B and C are shared with the XOR gate. The proposed QCA-based RUG gate requires 41 cells, an area of 37,604 nm^2^, and 0.75 clock cycles.

In the simulation results shown in Figure 6c, P has a high output polarization of 0.95, Q has 0.984, and R has 0.987. Although there is a slight jump in the output signal, it is very stable overall and there is no signal distortion.

**Figure 7:**
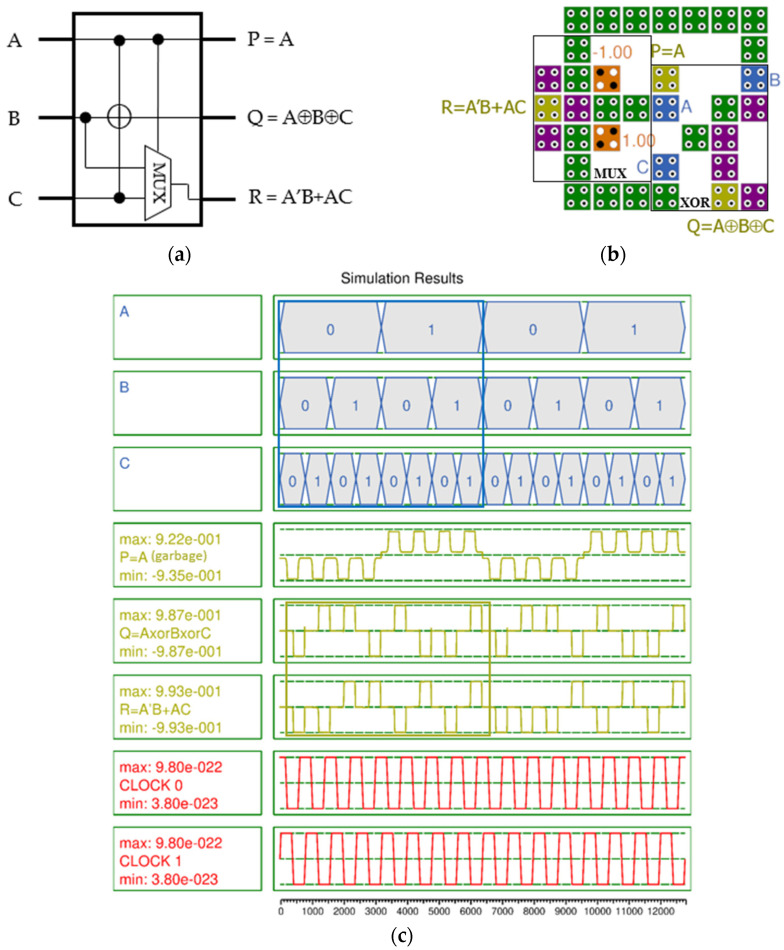
PQR gate with inputs (A, B, C) and outputs (P, Q, R): (**a**) logic diagram; (**b**) QCA implementation; (**c**) simulation results.

Figure 7a shows the logic diagram of the PQR circuit [10]. This circuit has the same output as the RQCA circuit except for the output of P, which is a garbage value and is needed to match the number of logical inputs and outputs of the reversible gate. Therefore, only an improved PQR circuit is proposed in this study. The second outputs the result of the XOR operation of the three inputs A, B, and C, and the third outputs A’B + AC, which is the result of a multiplexer with two inputs (B, C) and a selector A. For efficient design, a three-input XOR gate [24] was used, and a 2-to-1 Mux [25] that best matched the spatial arrangement was selected. The proposed PQR requires 35 cells, an area of 21,804 nm^2^, and 0.5 clock cycles.

The simulation results in Figure 7c show extremely high output polarization with valid output Q of 0.987 and R of 0.993. The output signal is very stable overall and does not show any signal jumping or distortion.

**Figure 8:**
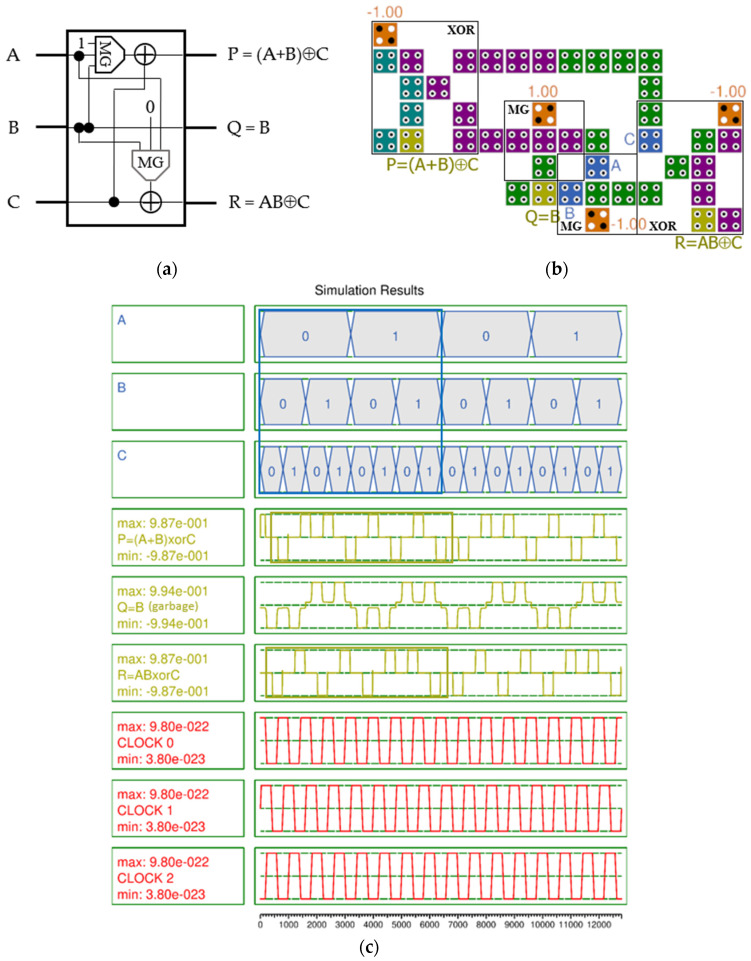
URG with inputs (A, B, C) and outputs (P, Q, R): (**a**) logic diagram; (**b**) QCA implementation; (**c**) simulation results.

Figure 8a shows the logic diagram of the URG using two majority voting gates and two two-input XOR gates, respectively [11]. The first and third outputs output (A + B)⊕C and AB⊕C, respectively, and the second has garbage output. For efficient design, two-input XOR gates were placed symmetrically on the left and right sides of the circuit, and two different majority gates were placed as close as possible to minimize area and delay, as shown in Figure 8b. The proposed URG requires 44 cells, an area of 43,924 nm^2^, and 0.75 clock cycles.

In the simulation results shown in Figure 8c, both the valid outputs P and R have a significantly high output polarization of 0.987. The output signal is very stable overall, and no signal jumping or distortion is observed.

## 4. Comparison and Analysis

In this section, we measure, compare, and analyze the performance of various previously proposed reversible gates and the current proposed structures. For accurate performance measurement and analysis, QCADesigner 2.0.3 [26] and QCADesingerE [27] were used. Related parameters were set as shown in Table 1.

Table 2 shows the performance and design costs for the proposed and existing circuits [10,11,28,29,30,31,32,33,34]. To compare performance, the numbers of cells, area, delay, and energy dissipation were measured, and two representative design costs were calculated. *Cell count* refers to the number of cells used for circuit design, *Area* refers to the rectangular area used for circuit design, *Delay* refers to the clock cycle required to obtain the result, and *E. D.* refers to the energy dissipation required for circuit operation. Energy dissipation is proportional to the area of the circuit and the density of cells, and a lot of heat is generated from the intersection and operation of the circuit. In order to minimize energy dissipation, the cells should be well distributed throughout the circuit to prevent cell bias, and the intersection of the circuit should be reduced [35,36,37].

*Cost_AD_* is calculated as $Area\times Delay^{2}$; time and area are included in the design cost, but this is a design cost calculation formula that places high emphasis on considering the importance of time [14,38]. *Cost_ED_* is calculated as ${E.D.}^{2}\times Delay^{2}$, and is a design cost calculation formula that takes time into consideration, as the importance of energy dissipation is evaluated highly [14,39]. Additionally, *AOP* stands for average output polarization and is a formula for measuring the strength of the output signal.

As shown in Table 2, the proposed QCA-based reversible gates showed significant superiority in most aspects of performance compared with existing circuits. Compared with existing gates, the proposed QCA-based TR circuit showed significant improvements of at least 45%, 63%, 50%, and 99% in cell numbers, area, delay, and energy dissipation, respectively. The proposed QCA-based RUG circuit showed significant improvements of 66%, 106%, 33%, and 21% in the mentioned performance indicators, respectively. For the QCA-based PQR circuit, the energy dissipation of the existing circuits was still low, but the proposed circuit showed meaningful improvements of 14%, 78%, and 50% in cell numbers, area, and delay, respectively. In addition, the proposed QCA-based URG circuit also showed excellent performance in terms of cell count, area, delay, and energy dissipation, showing improvements of 41%, 37%, 33%, and 66%, respectively, compared with the best existing circuit.

The superiority was clearly evident when comparing design costs. The proposed TR, RUG, PQR, and URG showed performance improvements of 266%, 265%, 300%, and 144% in *Cost_AD_*, respectively, compared with the best existing circuit. This shows outstanding improvement and superiority in terms of area and delay, which are the most important factors in circuit performance. The PQR circuit in [31] had the lowest E.D. due to its low cell density, resulting in the lowest related design cost. However, in *Cost_ED_*, the proposed TR, RUG, and URG showed tremendous improvements of 790%, 162%, and 389%, respectively. AOP is an important performance indicator that shows the qualitative stability of the circuit and ensures reliability for expansion and connection of the circuit. The proposed QCA-based reversible gates ensure very high and stable output signal strength.

## 5. Discussion

In this section, we discuss the simulation and evaluation methods in more depth. In this study, two design costs were treated as criteria for performance evaluation. This was in order to provide a clearer comparison by calculating the weight of each factor rather than simply comparing them using basic performances such as area, delay, and energy dissipation. Although there are various design cost calculation formulas, this study applied the most commonly used calculation formula for calculating circuit design cost [40,41,42]. In addition, AOP is directly related to the scalability of the circuit and is one of the important performance evaluation criteria that indicates reliability of the output signal. In addition, various qualitative evaluations such as the degree of output signal noise, circuit modularity, accessibility of input/output cells, and clock synchronization are required, but criteria that can objectify such qualitative evaluations are needed. Therefore, future studies are needed to establish criteria for qualitative and quantitative evaluations.

## 6. Conclusions

Efforts to protect information in quantum circuits have led to the development of various reversible gates, and many challenging studies have focused on quantum circuit design based on QCA to prevent information and energy loss. The current study optimized the performance of existing QCA-based reversible gates, and the excellence of the results was confirmed through significant improvement rates in various performance indices and design costs. In addition, the significant improvement in AOP is a meaningful result as this can increase the reliability of the circuit and enhance connectivity and scalability. It is expected that the optimization and weight reduction of reversible gates will greatly contribute to the development of nanocircuit design, which is becoming miniaturized.

## Figures and Tables

**Table 1:** Parameter settings on simulators.

Parameters	QCADesigner 2.0.3 Bistable Approximation	QCADesigner-E Coherence Vector with Energy
Cell size (nm)	18	18
Dot diameter (nm)	5	5
Cell separation (nm)	2	2
Layer separation (nm)	11.5	11.5
Clock high (J)	9.8 × 10^−22^	9.8 × 10^−22^
Clock low (J)	3.8 × 10^−23^	3.8 × 10^−23^
Clock shift	0	0
Clock amplitude factor	2.0	2.0
Relative permittivity	12.9	12.9
Radius of effect (nm)	65	80
Number of samples	12,800	-
Convergence tolerance	1.0 × 10^−3^	-
Maximum iterations per sample	100	-
Temperature (K)	-	1
Relaxation time (s)	-	1.0 × 10^−15^
Clock slope (s)	-	1.0 × 10^−12^
Time step (s)	-	1.0 × 10^−16^
Clock period (s)	-	4.0 × 10^−12^

**Table 2:** Performance comparison.

		Cell Count		Area		Delay		E. D.		Cost_AD_		Cost_ED_		AOP
Circuits		No.	Ratio	µm^2^	Ratio	Clocks	Ratio	10^−2^ eV	ratio	AD^2^	Ratio	E^2^D^2^	Ratio	
TR	[28]	116	4.00	0.3319	13.13	1.00	2.00	4.75	5.46	0.3319	52.54	22.56	119.24	9.37
	[29]	68	2.34	0.0737	2.92	1.00	2.00	3.33	3.83	0.0737	11.66	11.09	58.60	9.54
	[30]	225	7.76	0.4868	19.26	2.50	5.00	7.35	8.45	3.0425	481.56	337.64	1784.33	9.55
	[31]	42	1.45	0.0411	1.63	0.75	1.50	1.73	1.99	0.0231	3.66	1.68	8.90	9.48
	Figure 5b	29	1.00	0.0253	1.00	0.50	1.00	0.87	1.00	0.0063	1.00	0.19	1.00	9.87
RUG	[32]	106	2.59	0.1042	2.77	1.00	1.33	4.68	3.04	0.1042	4.93	21.90	16.42	9.38
	[33]	187	4.56	0.2034	5.41	1.25	1.67	6.08	3.95	0.3178	15.02	57.76	43.30	9.54
	[31]	68	1.66	0.0773	2.06	1.00	1.33	1.87	1.21	0.0773	3.65	3.50	2.62	9.51
	Figure 6b	41	1.00	0.0376	1.00	0.75	1.00	1.54	1.00	0.0212	1.00	1.33	1.00	9.74
PQR	[10]	90	2.57	0.0940	4.31	1	2.00	2.20	0.87	0.0940	17.24	4.84	3.05	9.53
	[31]	40	1.14	0.0388	1.78	0.75	1.50	1.41	0.56	0.0218	4.00	1.12	0.70	9.50
	Figure 7b	35	1.00	0.0218	1.00	0.5	1.00	2.52	1.00	0.0055	1.00	1.59	1.00	9.90
URG	[11]	134	3.05	0.1731	3.94	1	1.33	5.12	2.96	0.1731	7.01	26.21	15.57	9.53
	[34]	114	2.59	0.2211	5.03	1	1.33	4.91	2.84	0.2211	8.95	24.11	14.32	9.53
	[31]	62	1.41	0.0602	1.37	1	1.33	2.87	1.66	0.0602	2.44	8.24	4.89	9.48
	Figure 8b	44	1.00	0.0439	1.00	0.75	1.00	1.73	1.00	0.0247	1.00	1.68	1.00	9.87

## Data Availability

Data are contained within the article.
